# Discriminative ability of the original and short form of the Activities-specific Balance Confidence scale and its individual items for falls in people with multiple sclerosis

**DOI:** 10.1007/s13760-024-02515-y

**Published:** 2024-03-14

**Authors:** Zuhal Abasıyanık, Turhan Kahraman, Cavid Baba, Özge Sağıcı, Özge Ertekin, Serkan Özakbaş, on behalf of Multiple Sclerosis Research Group

**Affiliations:** 1 Department of Physiotherapy and Rehabilitation, Faculty of Health Sciences, Izmir Katip Celebi University, Izmir, Turkey; 2 Department of Health Professions, Faculty of Health and Education, Manchester Metropolitan University, Manchester, UK; 3 Graduate School of Health Sciences, Dokuz Eylül University, Izmir, Turkey; 4 Department of Neurological Physiotherapy-Rehabilitation, Faculty of Physical Therapy and Rehabilitation, Dokuz Eylül University, Izmir, Turkey; 5 Izmir University of Economics, Medical Point Hospital, Izmir, Turkey

**Keywords:** Multiple sclerosis, Falls, Balance confidence, Activities-specific Balance Confidence scale, Sensitivity, Specificity

## Abstract

**Background:**

Balance confidence is an essential component of fall risk assessment in persons with multiple sclerosis (pwMS).

**Aims:**

The aims of this cross-sectional study were to 1) investigate the ability of the 16-item Activities-specific Balance Confidence scale (ABC-16), 6-item Activities-specific Balance Confidence scale (ABC-6), and each item of the ABC-16 for distinguishing fallers and 2) determine cutoff scores for these scales to discriminate fallers and non-fallers in pwMS.

**Methods:**

One hundred and fifty-six participants [fallers/non-fallers: 60 (38.5%)/96 (61.5%), median EDSS: 1.5] were enrolled. Balance confidence was assessed using the ABC-16 and ABC-6. The self-reported number of falls in the past three months was recorded. Descriptive assessments, including walking, balance, and cognition were performed. Logistic regression and receiver operating characteristic analyses were conducted to estimate the sensitivities and specificities of the ABC-16 and ABC-6.

**Results:**

Both the ABC-16 (AUC: 0.85) and ABC-6 (AUC: 0.84) had the discriminative ability for falls. Each item of the ABC-16 scale was a significantly related to falls [odds ratio (OR) range: 1.38 to 1.89]. Items 8 and 10 had the highest odds ratio (OR: 1.85; 95%CI: 1.47–2.33, OR: 1.89; 95%CI: 1.49–2.40; respectively). We found cutoff scores of ≤ 70 of 100 (sensitivity: 71.67, specificity: 86.46) and ≤ 65/100 (sensitivity: 76.67, specificity: 79.17) in discrimination between fallers and non-fallers for the ABC-16 and ABC-6, respectively.

**Conclusion:**

Both original and short forms of the ABC scale are an efficient tool for discriminating fallers and non-fallers in pwMS. Although all items are related to falls, outdoor walking activities have the strongest associations with falls than other items.

## Introduction

Multiple Sclerosis (MS) is characterized by a wide range of complex symptoms that deteriorate physical, cognitive, and psychosocial functions [1]. It is well established that falls are an important and prevalent health concern in persons with MS (pwMS) [2]. Research consistently reports that over 50% of pwMS fall at least once in three months [3]. It is known that the risk of falling increases at the level of 4.0 and 6.0 disability scores measured by the Expanded Disability Status Scale (EDSS) and in the progressive type of MS [3]. However, even in non-disabled pwMS, the prevalence of fallers was found to be 25% [4]. Because of this high prevalence, it is important to examine the causes and consequences of falls in the MS population.

Accumulating evidence shows that falls are a multifactorial problem influenced by personal, physiological, and environmental factors, highlighting the personalized nature of fall management [2, 5]. Physiological factors, including balance impairment, muscle weakness, decreased walking speed, and use of mobility aids, have been associated with falls in pwMS [2, 6]. However, the ability of clinical balance and walking measures to identify falls is weak [7]. A recent review found that the predictive ability of the dual-task walking assessment for future falls is inconclusive [8]. Therefore, the influence of personal self-efficacy factors on falls has been investigated rather than relying solely on functional clinical measurements. Decreased balance confidence and increased fear of falling are shown as both causes and consequences of falls [9]. It has been shown that falls are mostly associated with balance confidence assessed by the Activities-specific Balanced Confidence scale (ABC), and the ABC is one of the best measurement tools for identifying fallers in MS and other populations [10–12].

The ABC is the most common patient-reported outcome measure used to assess balance confidence involving static, dynamic, proactive, and reactive balance insights [13]. It is a valid and reliable scale in the MS population [14]. However, to save time and to apply the ABC in busy clinical or research settings, the 6-item Activities-specific Balance Confidence scale (ABC-6) was obtained, and it demonstrated high validity for assessing balance confidence in pwMS [15, 16]. In studies including balance confidence assessment, the total score was presented, or the cutoff of the ABC scale has been used to discriminate between fallers and non-fallers [17, 18]. However, examining each item can provide quick screening for fall risk assessment because of their potential in healthcare decision-making for personalized assessment and treatment. Since the ABC scale is one of the most strongly associated tools for falls in pwMS and other populations [10, 11], specific items can provide invaluable insight into the falling status and behavior of pwMS, potentially allowing for individualized monitoring and training. To date, there is no study examining individual items of the ABC scale in pwMS. Furthermore, there is limited information about the discriminative ability of the ABC-6 in pwMS. For these reasons, our aims were to investigate the discriminative ability of both the original and short forms of the ABC scale, along with its individual items, in distinguishing between fallers and non-fallers in pwMS. Additionally, we aimed to determine cutoff scores with sensitivity and specificity values for both forms of the scale pwMS.

## Materials and methods

We performed a secondary analysis of the database of the study entitled, “Follow-up of physical, psychosocial and cognitive influences in persons with multiple sclerosis: a prospective cohort study” (ClinicalTrials.gov Identifier: NCT03878836). The institutional review board approval for this secondary analysis was obtained from the Noninvasive Research Ethics Board of XX University (Approval Number: 2021/22–37).

### Participants

Included participants had a clinically definitive diagnosis of MS, in the age range between 18 and 64 years, Expanded Disability Status Scale (EDSS) below 7 (with a median EDSS of 1.5, a minimum of 0, and a maximum of 6.5) and relapse-free within 30 days. The exclusion criteria were: 1) having a neurological disease other than MS, 2) having a musculoskeletal disorder that may influence gait and balance performance, and 3) having a severe cognitive impairment that prevented understanding of the assessments. The data were collected between January 2018 and February 2020. In total, there were 619 participants; however, only 156 pwMS who had complete data met the inclusion criteria for this study. All assessments were performed in a single session. Physical assessments were conducted by two physiotherapists, while the Symbol Digit Modalities Test (SDMT) was administered by a neuropsychologist.

### Primary outcome measures

#### The Activities-specific Balanced Confidence scale (ABC)

The ABC scale is a valid and reliable questionnaire that evaluates the level of confidence in performing a particular task without losing balance or becoming unsteady. Each of the 16 items is rated on a scale ranging from 0% (no balance confidence) to 100% (complete balance confidence). Averaging scores from all 16 items determine a total score [13]. Higher percentages reveal a higher level of balance confidence.

The ABC-6 includes the six most challenging tasks (items 5, 6, 13, 14, 15, and 16) of the ABC scale [15]. Balance confidence scores for the ABC-6 were calculated from the ABC scale.

#### Falls

In this study, we adopted the definition of a fall as “an event where the participant unintentionally landed on the ground or a lower level” [19]. Retrospective fall recall was collected by asking participants if they had fallen in the last three months. Participants in the fallers category were those reported at least one fall.

### Descriptive measures

Routinely collected data at the clinic were consolidated into this study’s data set, including the age, sex, EDSS score, disease duration, and type of MS.

*The Timed 25-foot Walk (T25FW)* test was used to assess the fastest walking speed. T25FW is one of the most common tests to quantify the fastest walking speed in the MS population and is a part of the MS Functional Composite disability assessment. It is applied in a 7.62 m walkway two times, and the average score is calculated [20]. *The Timed Up and Go test (TUG)* was used to evaluate mobility, transition, and balance with standard procedures in a 3 m walkway [21]. TUG was also performed under dual-task conditions. Participants performed counting backward in multiples of three from the starting number between 20 and 100. *The Six-Minute Walk Test (6MWT)* was used to assess the walking capacity. The participants were instructed to walk safely at their fastest speed in six minutes, according to the study of Goldman et al. The total distance in meters was recorded [22].

The static standing balance was evaluated using the *Single-Leg Stance Test (SLST).* Subjects were instructed to stand on their dominant feet with their eyes open without touching the opposite leg or ground for 60 s (seconds). The test ended when participants performed 60 s, or their feet touched the ground or their opposite extremities [23].

Cognitive processing speed and attention measurement were assessed using the *Symbol Digit modalities test (SDMT)*. The SDMT is a widely used, valid, and reliable neuropsychological tool for assessing cognitive deficits in the MS population [24, 25].

### Procedure

After the standard neurological examination of the people with MS at their routine clinical visits, they were assessed for the inclusion and exclusion criteria. First, basic demographics were recorded for the volunteer people, and the neuropsychologist applied SDMT. Then, the ABC scale was completed by the participants. Afterward, the SLST, T25FW, TUG, and 6MWT were administered with adequate rest intervals, respectively.

### Statistical analysis

IBM SPSS Statistics for Windows (Version 25.0. Armonk, NY: IBM Corp.), MedCalc Statistical Software (Version 15.8, MedCalc Software bvba, Ostend, Belgium) and MedCalc Software Ltd. Diagnostic test evaluation calculator (https://www.medcalc.org/calc/diagnostic_test.php, Version 20.015; accessed November 22, 2021) were used to analyze the data. Receiver operating characteristic (ROC) curve, area under the curve (AUC), sensitivity, specificity, Youden index J, positive and negative likelihood ratios (LR+ and LR−), and positive and negative predictive values (PPV and NPV), and test accuracy were calculated to investigate the discriminative ability of the ABC scale and its short form [26]. AUC is a global measure of diagnostic accuracy ranging from 0.5 (chance) to 1.0 (perfect accuracy). Values between 0.7 and 0.8 represent good diagnostic accuracy, 0.6 to 0.7 sufficient [27]. The false-positive (100 – specificity) and false-negative rates (100 – sensitivity) were calculated manually. The odds ratios (ORs) with 95% confidence intervals (95% CIs) were calculated for each item of the ABC scale for fall risk using the unadjusted logistic regression. Since higher ABC scale values indicate higher balance confidence, scores were inverted manually (100 – score) to calculate the ORs.

The Kolmogorov–Smirnov test, investigation histograms, and plots were used to check whether data were normally distributed. Differences between fallers and non-fallers in primary and descriptive measures were analyzed using a Chi-Squared test for categorical variables and by either independent t-test or Mann–Whitney U test, as appropriate, for continuous variables.

## Results

Demographic and clinical data are presented in Table 1. We analyzed the data of 156 participants. Sixty pwMS (38.5%) were fallers, and 96 (61.5%) were without a fall history. The EDSS was different between the groups (*p* < 0.001). Fallers reported significantly lower ABC-16 and ABC-6 scores than the non-fallers (*p* < 0.001). Additionally, the fallers showed significantly lower balance, walking, and cognitive performance in all clinical tests (*p* < 0.05).

**Table 1:** Descriptive values of demographic and clinical characteristics

	Total (*n* = 156)	Fallers (*n* = 60, 38.5%)	Non-fallers (*n* = 96, 61.5%)	*p*-value
Age (years), mean ± SD	36.57 ± 11.35	41.33 ± 10.63	33.59 ± 10.15	< 0.001
Gender, *n* (%)				0.714
Female	114 (73.1%)	45 (75%)	69 (71.9%)	
Male	42 (26.9)	15 (25%)	27 (28.1%)	
EDSS (0–10), median (25th–75th percentile)	1.5 (0–2.0)	2.0 (1.5–5.5)	1.0 (0–1.5)	< 0.001
MS course, *n* (%)				< 0.001
Relapsing–Remitting	143 (91.7%)	47 (78.3%)	96 (100%)	
Progressive	13 (8.3%)	13 (21.7%)	0 (0)	
Disease duration (years), median (25th–75th percentile)	6 (2–11.38)	8 (2.13–16.75)	5 (2–10)	0.016
Number of falls, median (25th–75th percentile)	0 (0–1)	1 (2–4)	N/A	N/A
ABC (0–100), mean ± SD	75.60 ± 23.53	57.87 ± 22.49	86.68 ± 16.33	< 0.001
ABC-6 (0–100), mean ± SD	67.63 ± 28.91	46.67 ± 26.63	80.74 ± 21.71	< 0.001
SLST (sec), mean ± SD	31.11 ± 24.85	19.42 ± 23.05	38.30 ± 23.23	< 0.001
T25FW (sec), mean ± SD	8.43 ± 20.37	13.69 ± 31.72	5.15 ± 4.88	< 0.001
TUG (sec), mean ± SD	9.19 ± 8.41	12.46 ± 10.09	7.26 ± 6.54	< 0.001
TUG-Cog (sec), mean ± SD	11.47 ± 11.65	15.55 ± 14.88	9.06 ± 8.42	< 0.001
6MWT (m), mean ± SD	429.58 ± 136.76	350.25 ± 168.07	478.33 ± 82.25	< 0.001
SDMT (score), mean ± SD	47.29 ± 12.41	43.93 ± 14.07	49.44 ± 10.77	0.01

*p < 0.05; *p*-value of independent t-test for continuous normal distributed data (mean ± SD), Mann–Whitney U test for non-normal distributed data [median (25th–75th percentile)], or chi-square for categorical variables

*SD* standard deviation; *EDSS* Expanded Disability Status Scale; *ABC* 16-item Activities-specific Balance Confidence; *ABC-6* 6-item Activities-specific Balance Confidence; *SLST* Single-Leg Stance Test; *T25FW* Timed 25-foot Walk; *TUG* Timed Up and Go; *TUG-Cog* Timed Up and Go test with cognitive task; *6MWT* Six-Minute Walk Test; *SDMT* Symbol Digit Modalities Test

**Fig. 1:**
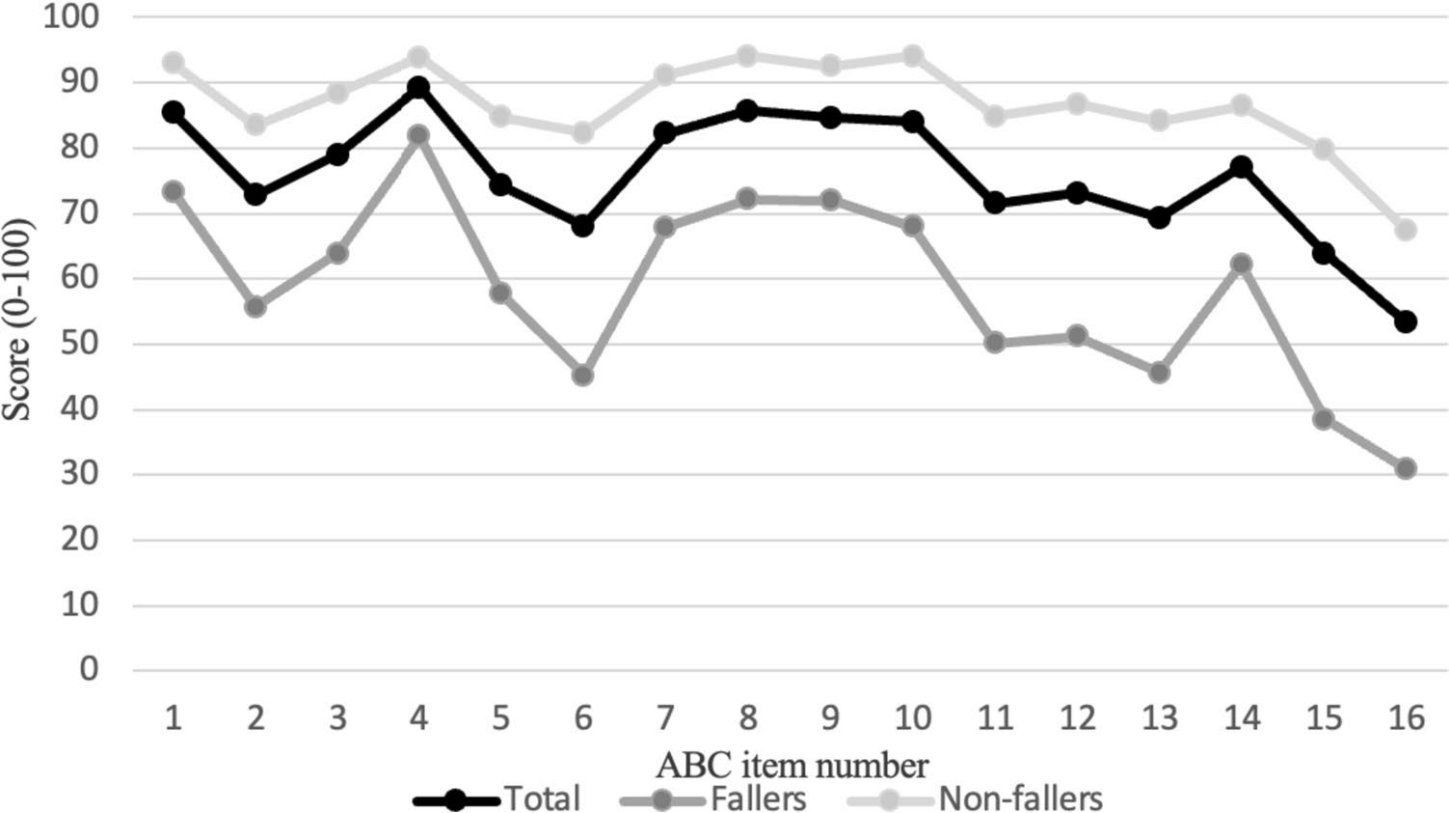
Mean scores of each item on the ABC-16 scale

Figure 1 shows the mean scores for each item in fallers, non-fallers, and all groups. The lowest score was in item 16 (walking outside on an icy sidewalk), and the highest was in item 4 (reaching for a small can off the shelf at eye level) for all groups.

**Fig. 2:**
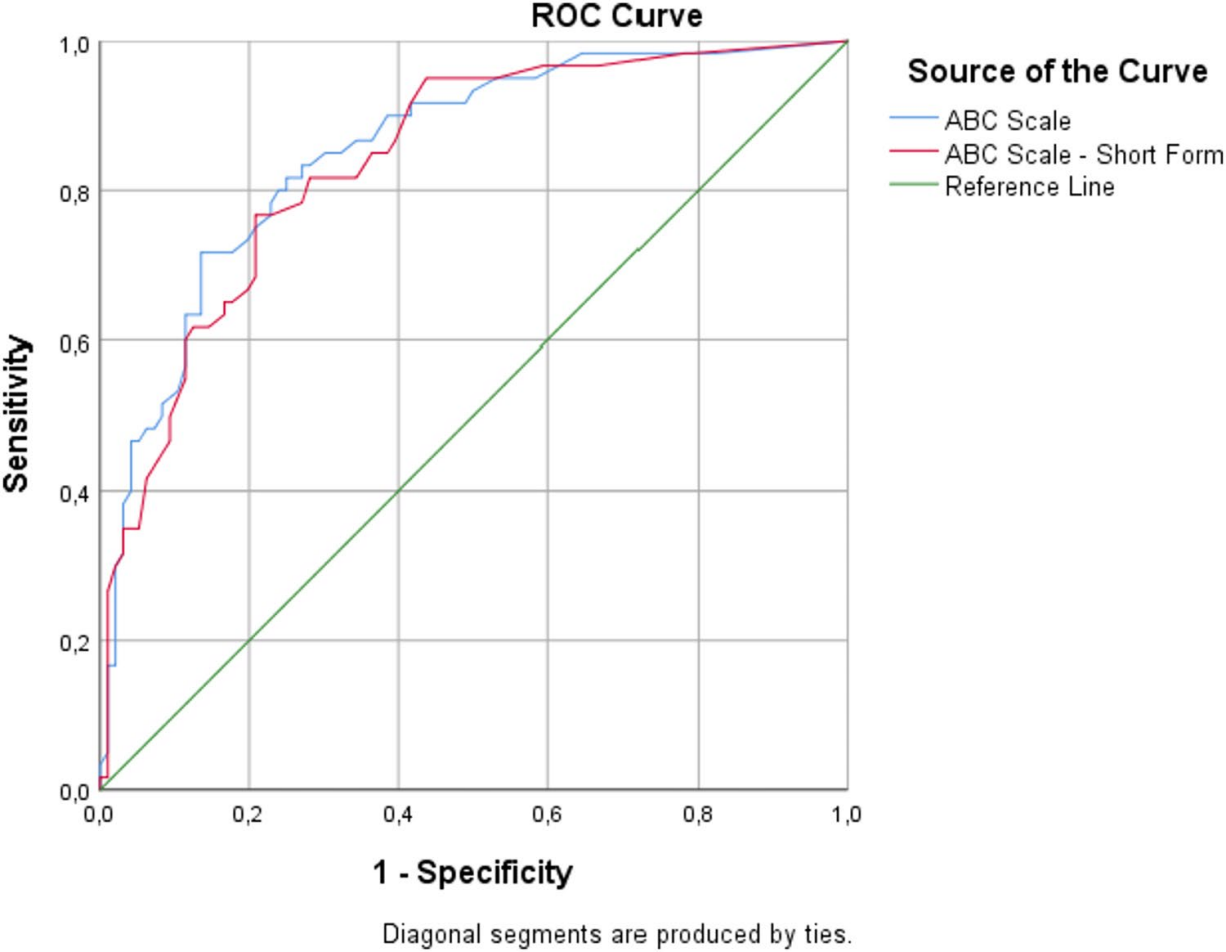
Receiver operating characteristic (ROC) curves of the ABC-16 and ABC-6 scales

Table 2 and Fig. 2 show the discriminative values of the ABC-16 and ABC-6 scales. Both the original and short forms showed good diagnostic ability. According to the AUC point estimator, the ABC-16 scale had slightly higher diagnostic accuracy than the ABC-6. The ROC analysis revealed a cutoff score is ≤ 70 for the ABC-16 scale and ≤ 65 for the ABC-6 scale. The ABC-6 scale has a slightly higher sensitivity compared to the ABC-16 scale. Both scales have a reasonably good ability to correctly identify fallers. Similarly, the ABC-16 scale has a slightly higher specificity compared to the ABC-6 scale. But the differences are not statistically significant based on the overlapping confidence intervals.

**Table 2:** Discriminative values of ABC-16 and ABC-6 scales

	ABC-16 scale	ABC-6
Cutoff score	≤ 70	≤ 65
Area under the curve (95% CI)	0.85 (0.79–0.91)	0.84 (0.77–0.89)
Youden index J	0.58	0.56
Sensitivity (95% CI), %	71.67 (58.6–82.5)	76.67 (64.0–86.6)
Specificity (95% CI), %	86.46 (78.0–92.6)	79.17 (69.7–86.8)
Positive likelihood ratio (95% CI)	5.29 (3.1–9.0)	3.68 (2.4–5.6)
Negative likelihood ratio (95% CI)	0.33 (0.2–0.5)	0.29 (0.2–0.5)
Positive predictive value (95% CI), %	76.79 (66.07–84.89)	69.70 (60.32–77.68)
Negative predictive value (95% CI), %	83.00 (76.41–88.04)	84.44 (77.24–89.68)
Test accuracy (95% CI), %	80.77 (73.70–86.63)	78.21 (70.90–84.41)
False-positive rate, %	13.54	23.33
False-negative rate, %	28.33	20.83

*CI* confidence interval

The discriminative ability of the individual items of the ABC-16 scale for falls is provided in Table 3. Univariate logistic regression analyses displayed that each item of the ABC-16 scale was significantly associated to falls in pwMS (OR range 1.38 to 1.89). The highest ORs were in item 10 (walk across parking lot to mall; OR: 1.89; 95%CI: 1.49–2.40) and item 8 (walking outside the house to a car parked in the driveway; OR: 1.85; 95%CI: 1.47–2.33). Item 16 has the lowest OR (walking outside on an icy sidewalk; OR: 1.38; 95%CI: 1.24–1.55).

**Table 3:** Univariate logistic regression models of individual items of ABC scale discriminating fall status

	Odds ratio (95% CI)	*p*-value
Item 1	1.74 (1.40–2.15)	< 0.001
Item 2	1.46 (1.27–1.68)	< 0.001
Item 3	1.53 (1.30–1.80)	< 0.001
Item 4	1.55 (1.23–1.96)	< 0.001
Item 5	1.43 (1.24–1.65)	< 0.001
Item 6	1.46 (1.29–1.66)	< 0.001
Item 7	1.58 (1.32–1.88)	< 0.001
Item 8	1.85 (1.47–2.33)	< 0.001
Item 9	1.65 (1.36–2.02)	< 0.001
Item 10	1.89 (1.49–2.40)	< 0.001
Item 11	1.56 (1.34–1.80)	< 0.001
Item 12	1.61 (1.38–1.88)	< 0.001
Item 13	1.57 (1.36–1.81)	< 0.001
Item 14	1.45 (1.25–1.68)	< 0.001
Item 15	1.45 (1.32–1.70)	< 0.001
Item 16	1.38 (1.24–1.55)	< 0.001

## Discussion

The primary purpose of this study was to determine the discriminative ability of the original and short forms of the ABC scale and its individual items, with the expectancy of accurately identifying fallers in pwMS. Our findings revealed that: 1) each item of the ABC scale was significantly associated with retrospective fall status in pwMS, 2) both short and original versions of the ABC scale can distinguish fallers and non-fallers, 3) the ABC-16 scale has slightly higher diagnostic accuracy than the ABC-6 for falls according to the AUC values, but both forms had good diagnostic accuracy, and 4) the cutoff score of the ABC-16 (≤ 70/100) showed moderate to good sensitivity and good specificity in differentiating of fallers and non-fallers. The cutoff score (≤ 65/100) of the ABC-6 showed good sensitivity and specificity.

Balance confidence assessed by the ABC has been considered an important determinant of falls in older people and people with neurological conditions [10, 28, 29]. In the study by Tajali et al., patient-reported outcomes (PROs) exhibited stronger associations with falls compared to clinical tests [11]. The ABC demonstrated the highest discriminative ability value among pwMS with mild to moderate disability, emphasizing its notable association with the occurrence of falls [11]. Similarly, the ABC has the highest explanatory value for falls in pwMS without a neurological disability [4]. The fact that most of the participants in this study were mildly disabled and the ABC scale had a high identification ability for fallers supports these results. Furthermore, in line with the previous studies, the ABC scores differed significantly between fallers and non-fallers (6) [17]. There is only one study on the ability of ABC-6 to discriminate between fallers and non-fallers [30]. Wood et al. found that although ABC-6 had poor sensitivity and moderate specificity in discriminating fallers, there is good sensitivity and specificity for distinguishing high fall-risk groups according to the Physiological Profile Assessment. Our findings suggested that the ABC-6 had good identification ability according to the AUC and good sensitivity and specificity values. Thus, clinicians and researchers can prefer the ABC-6 to save time or reduce the burden of evaluation for patients.

As mentioned above, growing evidence suggests that the ABC is strongly related to falls. This finding may be related to the fact that the ABC items query static, dynamic, proactive, and reactive balance in indoor and outdoor conditions. Hence, understanding which condition is more strongly associated with falls could serve as a valuable indicator in clinical settings to guide rehabilitation strategies. In our study, each item significantly associated with falls. However, the highest association was observed in the items related to the outdoor walking activities (Items 8 and 10). Considering the evidence that most falls occur indoors [3], the higher discriminative ability of falls in outdoor walking activities in this study may be associated with the participants having lower levels of disability and being younger [31]. The fact that the OR was lower in items such as items 6, 15, 16, which scored the lowest by both fallers and non-fallers suggests that the challenging activities were less discriminative even though the disability level was low. This result confirms the previous findings that falls generally occur in more general mobility activities [2].

To our knowledge, there is no study for determining cutoff scores of ABC-16 and ABC-6 in pwMS. In some studies, the study of Dibble et al. performed in 61 pwMS, and the mean ABC score of fallers, 63.92, was used to categorize pwMS according to fall risk [17]. Although the mean ABC score of fallers in our study (57.87) was lower than the finding of Dibble et al., the cutoff score we found (≤ 70 for the ABC-16; ≤ 65 for the ABC-6) with the appropriate analyses is lower. The cutoff score we found in our study is lower than the cutoff scores defined in individuals with Parkinson’s disease (≤ 77.5 for the ABC-16; ≤ 65.8 for the ABC-6 [28]. The lower cutoff and total score of ABC-6 than the ABC-16 in the study of Cole et al. [28] and our study support the notion that the ABC-6 includes the most challenging activities of the ABC-16 scale.

It is important to note that the generalizability of the findings of our study may be limited because most of our participants had a mild disability. However, the changing profile of pwMS and the increase in the number of patients with an EDSS score of ≤ 3 in recent years may not limit the applicability of our findings in the clinical setting [32]. The lower level of disability and the retrospective fall recall may have resulted in reporting a lower prevalence of falls compared to studies in the literature that assess falls over the last 6 months. The cross-sectional design limits the establishment of a cause-and-effect relationship. Additionally, the authors did not control the data collection procedure, which is the nature of archival data secondary analyses. Lastly, regression analyses were not adjusted, which underscores the importance of considering potential confounding variables for a more nuanced interpretation of the results.

## Conclusion

Our results support the use of both the ABC-16 and ABC-6 scales to identify fallers in the MS population. While the ABC-16 scale assesses various aspects of balance, our findings suggest a stronger association between outdoor walking and falls. Scores of ≤ 70/100 and ≤ 65/100 can be used as cutoffs to classify pwMS as fallers and non-fallers using the ABC-16 and ABC-6 scales, respectively. It would be beneficial for future studies to collect longitudinal data to further enhance our understanding of the predictive capabilities of both the ABC-16 and ABC-6 scales in identifying fallers in pwMS.

## Data Availability

The data that support the findings of this study are available from the corresponding author upon relevant request.
